# Identification of polycystic ovary syndrome potential drug targets based on pathobiological similarity in the protein-protein interaction network

**DOI:** 10.18632/oncotarget.9353

**Published:** 2016-05-13

**Authors:** Hao Huang, Yuehan He, Wan Li, Wenqing Wei, Yiran Li, Ruiqiang Xie, Shanshan Guo, Yahui Wang, Jing Jiang, Binbin Chen, Junjie Lv, Nana Zhang, Lina Chen, Weiming He

**Affiliations:** ^1^ College of Bioinformatics Science and Technology, Harbin Medical University, Harbin, Heilongjiang Province, China; ^2^ Department of Endocrinology and Metabolism Laboratory, The Second Affiliated Hospital of Harbin Medical University, Harbin, Heilongjiang Province, China; ^3^ Institute of Opto-electronics, Harbin Institute of Technology, Harbin, Heilongjiang Province, China

**Keywords:** polycystic ovary syndrome, protein-protein interaction network, pathobiological similarity, module, drug target

## Abstract

Polycystic ovary syndrome (PCOS) is one of the most common endocrinological disorders in reproductive aged women. PCOS and Type 2 Diabetes (T2D) are closely linked in multiple levels and possess high pathobiological similarity. Here, we put forward a new computational approach based on the pathobiological similarity to identify PCOS potential drug target modules (PPDT-Modules) and PCOS potential drug targets in the protein-protein interaction network (PPIN). From the systems level and biological background, 1 PPDT-Module and 22 PCOS potential drug targets were identified, 21 of which were verified by literatures to be associated with the pathogenesis of PCOS. 42 drugs targeting to 13 PCOS potential drug targets were investigated experimentally or clinically for PCOS. Evaluated by independent datasets, the whole PPDT-Module and 22 PCOS potential drug targets could not only reveal the drug response, but also distinguish the statuses between normal and disease. Our identified PPDT-Module and PCOS potential drug targets would shed light on the treatment of PCOS. And our approach would provide valuable insights to research on the pathogenesis and drug response of other diseases.

## INTRODUCTION

Polycystic ovary syndrome (PCOS) is one of the most common endocrinological disorders in reproductive aged women, of which the prevalence is 5–10% in the general population [1–3]. The clinical and biochemical characteristics of PCOS are generally grouped into two main categories: (i) reproductive features that mainly include chronic anovulation, and (ii) hyperandrogenic features that mainly include hyperandrogenaemia.

As important metabolism characteristics, insulin resistance and compensatory hyperinsulinemia are observed in approximately 70% of PCOS women [4], which contribute to the strong association of PCOS with adverse metabolic risk, including dysglycemia, dyslipidaemia and fatty liver, as well as increase the risk for development of type 2 diabetes (T2D) [5]. It is found that some other features including weight gain, disturbance of hormones and lipid disorders are associated with both PCOS and T2D [6, 7]. In the meantime, PCOS and T2D are closely linked in the epidemiology and etiology as well as in the systems biology [8, 9]. Significant pathobiological similarity between PCOS and T2D was also indicated (−0.2) after determining the pathobiological and clinical relationship between these diseases using a method proposed by Jörg Menche et al. [9].

Metformin is the only known drug for PCOS in Drugbank. The DrugBank database is a unique bioinformatics and cheminformatics resource that combines detailed drug data with comprehensive drug target information, which was widely recognized and used [10]. As an insulin sensitizer, Metformin can cause reductions in body weight, restore ovulation, increase the rate of pregnancy and reduce the number of pregnancy complications [11]. In the meantime, metformin is also used for treating T2D, which improves glycemic control by decreasing hepatic glucose production and glucose absorption and increasing insulin-mediated glucose uptake [12]. In addition, Thiazolidinediones (TZDs), including pioglitazone, are peroxisome proliferator-activated receptor (PPAR) agonists that induce adipogenesis and have insulin-sensitizing and antidiabetic properties [13]. Recent studies have demonstrated the beneficial metabolic effects of treatment with pioglitazone in PCOS patients [11, 14]. These indicated that the genes and drugs associated with common metabolism characteristics of PCOS and T2D might be the potential drug targets and drugs of PCOS.

The research on complex diseases from the systems level and biological background might provide new insights into drug target identification, disease gene exploration and pathogenesis elucidation [15–17]. For example, disease genes and drug targets of coronary heart disease (CAD) and T2D could be identified effectively by systems biology approaches [18, 19]. Chan KH et al. identified key drivers that included COL1A1, COL3A1, and ELN in the shared pathways for both CAD and T2D based on the protein-protein interaction network (PPIN) and genome-wide association studies [20]. In addition, the highly central importance proteins in core networks or sub-networks are globally or locally important nodes which could act as functional modules or pathways or key role genes to uncover the pathogenesis and therapeutic ways of disease [21]. For example, Li H et al. presented a novel PPI knowledge-based approach that identified functional modules and hub genes for CAD, revealing several novel pathogenic mechanisms [20]. Wan Li et al. developed a method using combined centrality indices on the systems level to identify essential cancer-related motifs in central roles, which provided a clue for the study of signal transduction in biological pathways [22]. Furthermore, Emad Fadhal et al. have indicated that the genes possessing highly topologically centered in the PPIN may be good therapeutic targets [23].

**Figure 1:**
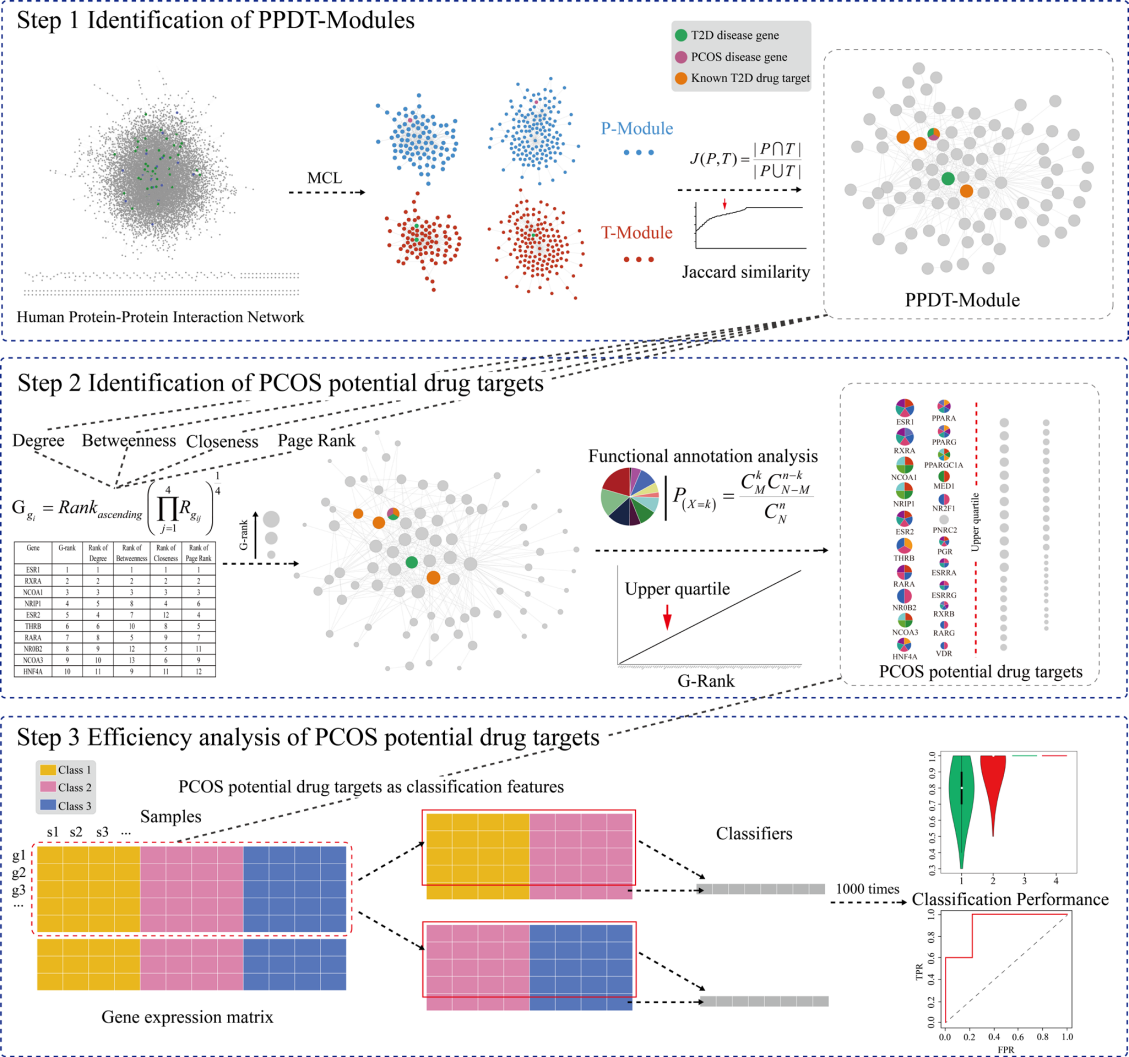
A schematic diagram of PCOS potential drug targets identification and efficiency analysis

In this study, based on the pathobiological similarity of PCOS and T2D, a new computational approach (Figure 1) was performed to identify the PCOS potential drug target modules (PPDT-Modules) and PCOS potential drug targets from the systems level and biological background in the PPIN. Our study may reveal novel PCOS therapeutic ways and improve the cure rate of PCOS.

## RESULTS

### PPDT-Modules

The PPDT-Module identification approach we put forward was performed with 35 PCOS disease genes, 83 T2D disease genes and 62 T2D drug targets in the PPIN. 910 P-Modules (13 P-Modules contained one or more PCOS disease genes) and 923 T-Modules (25 T-Modules contained one or more T2D disease genes) were identified. The Jaccard similarity index was calculated for each P-Module and T-Module pairs. 3 candidate PPDT-Modules were identified ultimately, including PCOS disease genes, T2D disease genes and known T2D drug targets simultaneously (Table 1, Supplementary Table 1).

**Table 1:** Candidate PPDT-Modules

Candidate PPDT-Module	The number of genes in PPDT-Module	PCOS disease Gene	T2D disease Gene	T2D drug Target
Candidate PPDT-Module 1	141	TH	UCP3	PTPN1, ADRA2C
Candidate PPDT-Module 2	85	PPARG	HNF4A, PPARG	NCOA1, PPARA PPARD, PPARG
Candidate PPDT-Module 3	6	INSR	INSR, ENPP1	INSR

### Central importance of genes in PPDT-Modules

Degree, closeness, betweenness, and page rank of genes in PPDT-Modules were calculated, and the genes in each module were ranked by these topological metrics, respectively. The G-rank of genes in PPDT-Modules was calculated using the geometric mean rank (G- rank of top 22 genes and all genes in PPDT-Module 2are shown in Table 2 and Supplementary Table 2, respectively).

**Table 2:** G-rank of top 22 genes in PPDT-Module 2

Gene	G-rank	Rank of Degree	Rank of Betweenness	Rank of Closeness	Rank of Page Rank
ESR1	1	1	1	1	1
RXRA	2	2	2	2	2
NCOA1	3	3	3	3	3
NRIP1	4	5	8	4	6
ESR2	5	4	7	12	4
THRB	6	6	10	8	5
RARA	7	8	5	9	7
NR0B2	8	9	12	5	11
NCOA3	9	10	13	6	9
HNF4A	10	11	9	11	12
PPARA	11	12	4	30	10
PPARG	12	7	11	28	8
PPARGC1A	13	13	22	7	13
MED1	14	14	20	10	14
NR2F1	15	28	6	13	24
PNRC2	16	15	19	16	17
PGR	17	19	14	22	15
ESRRA	18	18	21	14	19
ESRRG	19	16	23	39	16
RXRB	20	17	35	27	18
RARG	21	20	26	33	23
VDR	22	21	25	36	21

The PPDT-Modules were the pathobiological similar modules of PCOS and T2D. The higher genes of PPDT-Module were ranked, the more important they might be, and the more kernel roles they would act in the pathogenesis and treatment of PCOS. The genes ESR1, RXRA and NCOA1 were the top three genes not only in the G-rank of genes, but also in four individual topological ranks, which demonstrated the stability of PPDT-Module 2. It was noted that ESR1 (1st) and ESR2 (5th) both ranked highly in the G-rank of genes in PPDT-Module 2. They are the main receptors of estrogen and the metabolism of estrogens was proved to be associated with the pathogenesis of PCOS [24]. The gene RXRA (2nd) is always binding with PPARG and function together, and the gene PPARG is both the PCOS disease gene and T2D disease gene, is also the known T2D drug target, and ranked 12th in PPDT-Module 2. The gene NCOA1 (3rd) is one of the known T2D drug targets. In addition, the gene PPARA, ranked 11th in PPDT-Module 2, is one of the known T2D targets. So genes which possessed high G-rank in PPDT-Module might play key roles in the pathogenesis, diagnosis and treatment of PCOS.

### PCOS potential drug targets

**Figure 2:**
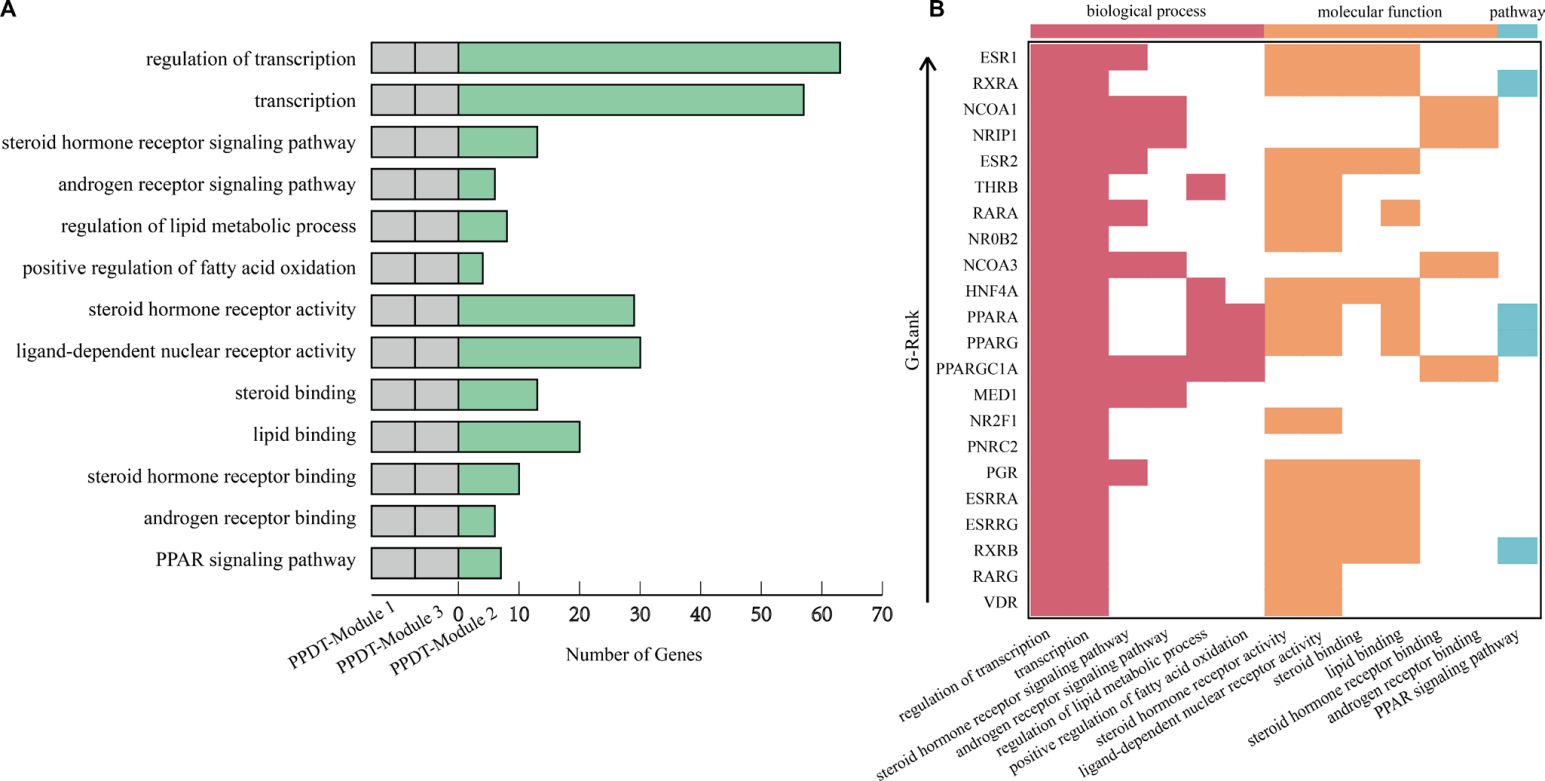
Main functions annotated by PPDT-Modules (**A**) Main functions annotated by 3 candidate PPDT-Modules and PCOS disease genes. The first two columns of gray bars represent no gene of candidate PPDT-Module 1 and candidate PPDT-Module 3 enriched significantly in corresponding functional categories, respectively. The third column of green bars represents the number of genes of candidate PPDT-Module 2 enriched in the functional categories. (**B**) Main functions annotated by genes in PPDT-Module 2. Main functions annotated by genes in PPDT-Module 2. Each row represents a gene in PPDT-Module 2, and each column represents a functional category: biological process, molecular function and pathway. Arrow represents the ascending trend of G-Rank of genes in PPDT-Module 2.

From the functional perspective to analyze the association between PPDT-Modules and PCOS, the hyper-geometric test was performed with the genes in PPDT-Modules and the PCOS disease genes in the Gene Ontology (GO) and pathways of Kyoto Encyclopedia of Genes and Genomes (KEGG), and the p value was adjusted with FDR (*P* < 0.05, Figure 2A).

The genes of PPDT-Module 1 and PCOS disease genes, PPDT-Module 3 and PCOS disease genes are not enriched significantly in the functional categories related to PCOS, respectively (Figure 2A). The genes of PPDT-Module 2 and PCOS disease genes are enriched significantly in functional categories and KEGG pathways associated with the pathogenesis of PCOS (Figure 2A, Supplementary Table 3), such as transcription, steroid hormone receptor signaling pathway (GO biological process, BP), steroid binding, lipid binding and steroid hormone receptor binding (GO molecular function, MF), PPAR signaling pathway, Steroid hormone biosynthesis (KEGG pathway).

“GO:0030518~steroid hormone receptor signaling pathway”, “GO:0003707~steroid hormone receptor activity”, “GO:0005496~steroid binding” and “GO:0005496~steroid binding” are associated with steroid hormones. Disturbed steroid hormone receptor signaling pathway would lead to the hormonal imbalance which is a main underlying problem with PCOS [25]. The steroid hormones mainly contain the androgens and estrogens and so on. “GO:0030521~androgen receptor signaling pathway” and “GO:0050681~androgen receptor binding” are also associated with androgen. Androgen receptor signaling is an important mediator in the etiology of PCOS traits [26]. And androgens mediate their steroidal actions via the androgen receptor which are crucial in maintaining female fertility by optimizing follicle growth, health, and ovulation [27]. With PCOS, women typically have high levels of androgens, high levels of androgens affect the development and release of eggs during ovulation [27–29]. “GO:0030520~estrogen receptor signaling pathway” and “GO:0030331~estrogen receptor binding” are associated with estrogen. It is noted that estrogens play important roles in the development and functioning of the male and female reproductive systems [30, 31]. The main mediators of estrogen action are 2 specific high-affinity receptors, the ESR1 and ESR2, are necessary for the proper function of the hypothalamic–pituitary–ovarian axis and are expressed in the human ovary, where ESR2 is the predominant receptor and its activation enhances folliculogenesis and ovulation [32, 33]. The expression of ESR2 is lower in follicles derived from women with PCOS compared with healthy women, while ESR1 expression is markedly increased in theca cells of polycystic ovaries, causing alteration in the ESR1/ESR2 ratio in PCOS and possibly abnormal follicular development [34, 35]. Otherwise, insulin resistance is associated not only with the pathogenesis of T2D, but also with the pathogenesis of PCOS [36, 37]. The role of ESR1 in the development of insulin resistance has been described in human with a null mutation in the ESR1, leading to unresponsiveness to estrogen, who developed impaired glucose tolerance [34, 38].

“GO:0019216~regulation of lipid metabolic process”, “GO:0004879~ligand-dependent nuclear receptor activity” and “GO:0008289~lipid binding” are associated with lipid metabolism. Abnormal lipid metabolism is one of the main metabolic characteristics of PCOS patients [39]. The parameters of lipid metabolism were significantly different in women with PCOS from in healthy women, higher triglycerides (TG), and very-low-density lipoprotein cholesterol (VLDL) with lower high-density lipoprotein cholesterol (LDL) in PCOS [40, 41]. Altered expression of genes involved in lipid metabolism could contribute to insulin resistance in non-obese PCOS patients [39]. Based on case-controlled studies, women with PCOS appear to have a high prevalence of the metabolic syndrome including obesity and dyslipidemia, and have a higher risk of diabetes [2]. Insulin resistance appears to have a pivotal role with multiple studies demonstrating significant associations between altered lipid metabolism and insulin level, which may be induced in part by the insulin-medicated stimulation of lipolysis and altered expression of lipoprotein lipase and hepatic lipase [42, 43]. Some studies indicated that disturbance of transcription, disturbance of steroid hormone, lipid abnormalities and so on should lead to promoting the PCOS [44–46].

Meanwhile, “hsa03320:PPAR signaling pathway” plays an important role in the pathogenesis of PCOS and T2D, and the treatment for T2D. Peroxisome proliferator-activated receptors (PPARs) are nuclear hormone receptors activated by fatty acids and their derivatives [13]. PPAR signaling pathway was shown to associate with the pathogenesis of PCOS, as well as with the pathogenesis of T2D [13]. PPAR has three subtypes (PPARA, PPARD, and PPARG), all of them are known T2D drug targets, are showing different expression patterns. PPARA plays a role in the clearance of circulating or cellular lipids via the regulation of gene expression involved in lipid metabolism in liver and skeletal muscle [47]. PPARD is involved in lipid oxidation and cell proliferation [47, 48]. PPARG promotes adipocyte differentiation to enhance blood glucose uptake [49]. Meanwhile, PPARG is both PCOS and T2D disease gene, the drug targeting to PPARG was used to treat with PCOS patient and had good curative effect [13].

**Figure 3:**
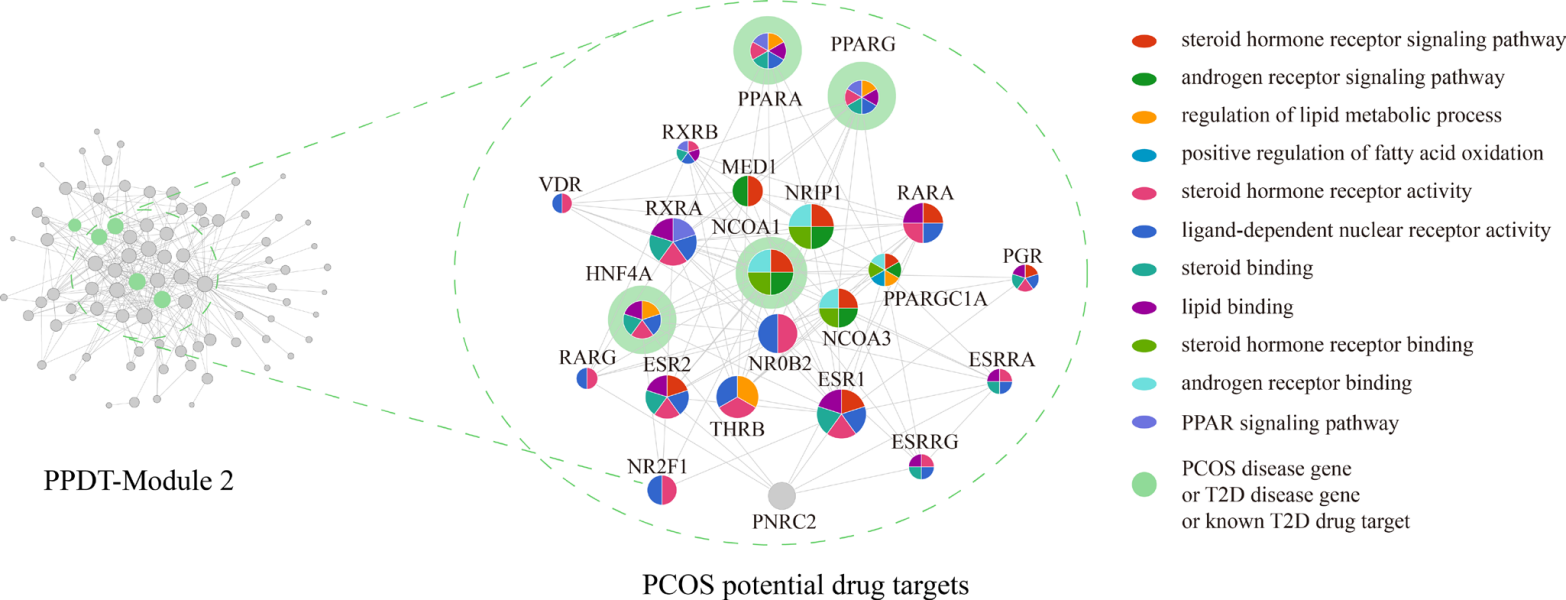
PPDT-module 2 and PCOS potential drug targets The size of nodes represents their G-Ranks. Different colors represent different functional categories. The green circle represents PCOS disease gene or T2D disease gene or known T2D drug target.

These changes of function and disturbance of pathways were associated not only with PCOS, but also with T2D closely. So, the genes of PPDT-Module 2 enriched significantly in these functions, may act as PCOS and T2D disease genes and drug targets (Figure 2B), such as PPARG. Known T2D drug targets NCOA1 and PPARA, T2D disease genes HNF4A, and the PCOS disease genes AR, CYP11A1 were also enriched in same functional categories and pathways of PPARG. Meanwhile, these disease genes and drug targets in PPDT-Module 2 had a high G-Rank, for instance, NCOA1 ranked 3rd, HNF4A ranked 10th, PPARA ranked 11th, PPARG ranked 12th. Hence, 22 genes of PPDT- Module 2 whose G-Rank was the upper quartile as well as enriched significantly in these PCOS associated functional categories and pathways were identified as the PCOS potential drug targets (Figure 3, Table 2).

### Efficiency analysis of PCOS potential drug targets

**Figure 4:**
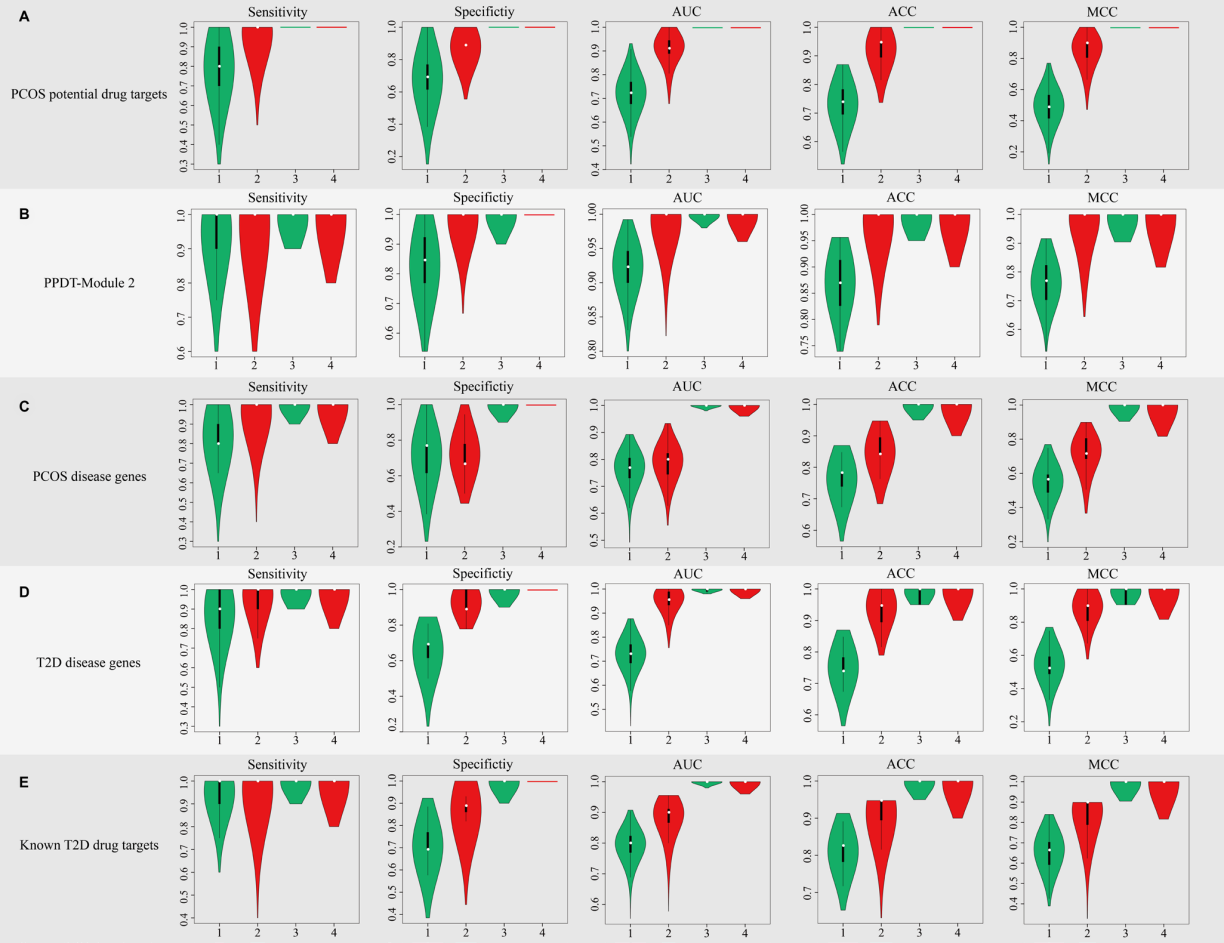
The sensitivity, specificity, AUC, ACC and MCC score distribution of different classification features with 1000 times five-fold cross-validation In each figure, the first two boxes represent the distribution of classification of normal/PCOS samples, the next two represent the distribution of classification of PCOS/after pioglitazone treatment samples. Green represents the classification of the samples before consistency check, red represents the classification of the samples after consistency check.

**Figure 5:**
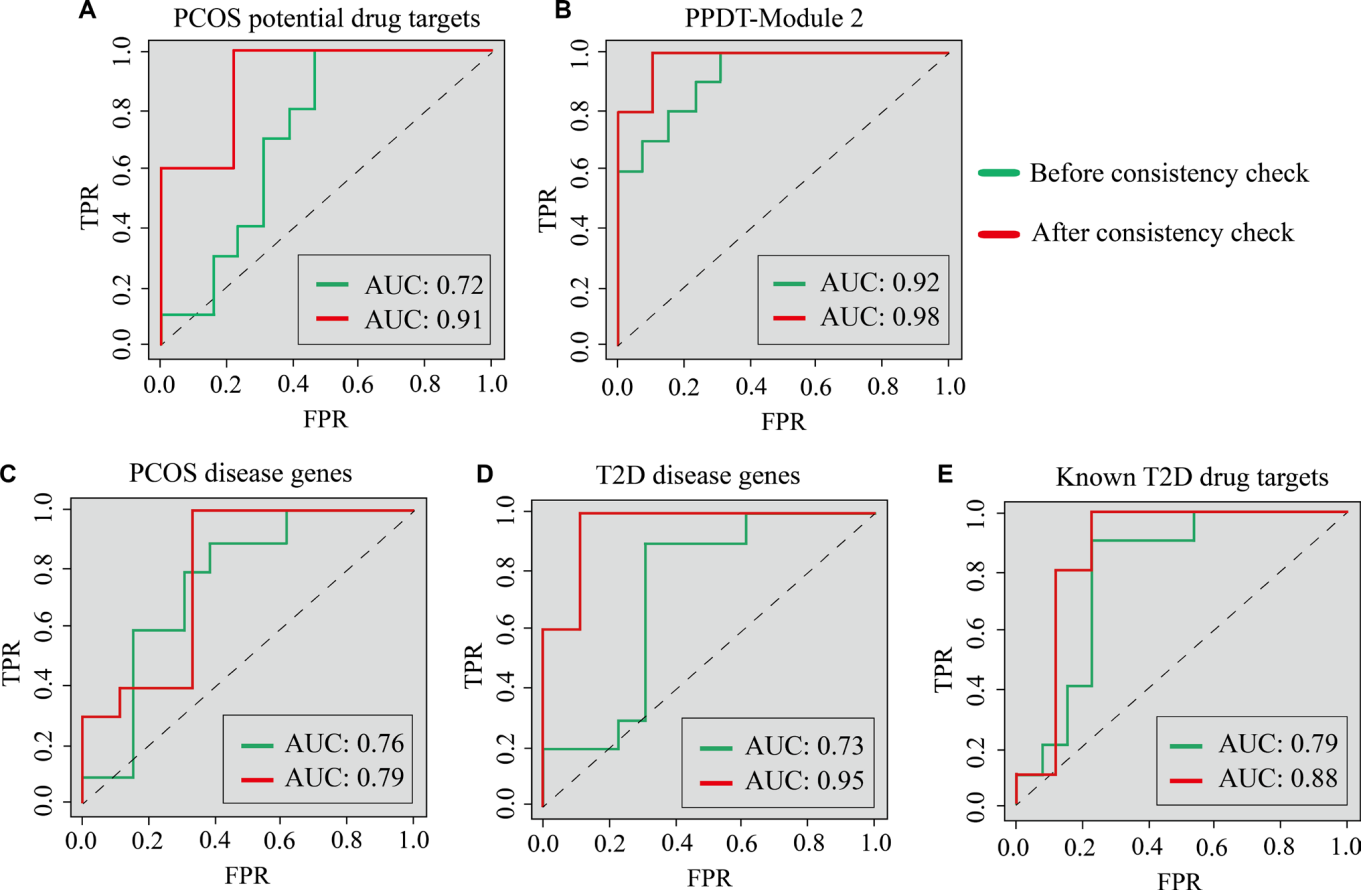
The ROC curves of classification for normal/PCOS with different features ROC curves of classification for normal/PCOS with (**A**) PCOS potential drug targets, (**B**) PPDT-Module 2, (**C**) PCOS disease genes, (**D**) T2D disease genes and (**E**) Known T2D drug targets as classification features, respectively. Green lines represent the ROC curves before consistency check, red lines represent the ROC curves after consistency check.

To assess the efficiency of PCOS potential drug targets, SVM was applied to classify samples of different statuses (normal/PCOS, PCOS/after pioglitazone treatment) of GSE8157 with the average expression values of identified PCOS potential drug targets as classification features. 1000 times of five-fold cross-validation were performed (Figure 4). With the average expression values of 22 PCOS potential drug targets as the classification features, the average of sensitivity values for normal/PCOS, PCOS/after pioglitazone treatment were 0.79 and 1, the average of specificity values were 0.68 and 1, the average of AUCs were 0.72 and 1, the average of accuracy values were 0.73 and 1, the average of MCC scores were 0.49 and 1, respectively (Figure 4, Figure 5A, Supplementary Table 5, and Supplementary Table 6). At the same time, the average expression values of PCOS disease genes, T2D disease genes and known T2D drug targets were used as the classification features to classify the same samples of different statuses, respectively (Figure 4, Supplementary Table 5, and Supplementary Table 6). Most of these classification features could classify samples efficiently.

For the original expression profile, expression consistency check was performed via Wilcoxon signed-rank test to test the sample consistency of the same status (13 normal samples, 10 PCOS samples and 10 after pioglitazone treatment samples, FDR *P* value < 0.05), respectively. 9 of 13 normal samples and 5 of 10 after pioglitazone treatment samples were consistent. These 5 samples may alter to a common status after drug treatment, suggesting similar drug response and good curative effect of drug treatment, so the paired 5 PCOS/after pioglitazone treatment samples were selected and a new SVM was applied to classify the consistent samples of 3 statuses using the above-mentioned classification features (Figure 4). With identified PCOS potential drug targets as classification features, the average of sensitivity values for normal/PCOS, PCOS/after pioglitazone treatment were 0.95 and 1, the average of specificity values for normal/PCOS, PCOS/after pioglitazone treatment were 0.88 and 1, average of AUC values of normal/PCOS and PCOS/after pioglitazone were 0.91 and 1, the average of accuracy values for normal/PCOS, PCOS/after pioglitazone treatment were 0.92 and 1, the average of MCC scores for normal/PCOS, PCOS/after pioglitazone treatment were 0.84 and 1, respectively (Figure 4, Figure 5A, Supplementary Table 5, and Supplementary Table 6). The classification performance of consistent samples was much better than that of all samples from the original profile with the same classification features, especially for using the average expression values of identified PCOS potential drug targets and known T2D disease genes as classification features (Figure 4, Figure 5, Supplementary Table 5, and Supplementary Table 6), which demonstrated the effectiveness of identified PCOS potential drug targets. PCOS potential drug targets could effectively reflect drug response, classify the samples between normal and disease and between disease and after drug treatment, as well as represent the treatment effect to PCOS accurately. Interestingly, identified PCOS potential drug targets had a better classification performance than known PCOS disease genes, which demonstrated that these potential drug targets may act as the PCOS disease genes (Figure 4, Figure 5, Supplementary Table 5, and Supplementary Table 6). Classification using identified PPDT-Module 2 as classification features had good performance for normal/PCOS and for PCOS/after pioglitazone treatment in both original and consistent samples (Figure 4B, Figure 5B, Supplementary Table 5, and Supplementary Table 6).

## DISCUSSION

PCOS is a disorder of irregular menses, hyperandrogenism and/or polycystic ovary morphology [26]. A large proportion of women with PCOS also exhibit insulin resistance, β cell dysfunction, impaired glucose tolerance and/or T2D [50]. Women with T2D have a higher prevalence of polycystic ovary syndrome compared to the general population [8]. There is also a high rate of T2D in family members of women with PCOS [50]. Some studies have demonstrated that there are common biological background and marker between PCOS and T2D [51]. Based on these, we put forward a new method to identify 3 candidate PPDT-Modules from the systems level in the PPIN based on the pathobiological similarity of PCOS and T2D. Identified PPDT-Modules contained many PCOS and T2D disease genes, and known T2D drug targets, simultaneously. By the functional annotation analysis with the PCOS disease genes, candidate PPDT-Module 2 was identified as the final PPDT-Module. The functional categories and pathways that genes of PPDT-Module 2 and PCOS disease genes enriched significantly, are associated not only with pathogenesis and treatment of T2D, but also with PCOS. Considering the central importance and functional annotation of known disease genes and targets, 22 PCOS potential drug targets were identified. These PCOS potential drug targets could describe the drug treatment effect well by an independent profile verified, and the 21 genes (95.45%) had been verified by literature to be associated with the pathogenesis and treatment of PCOS (Supplementary Table 4).

Dysfunctions of steroid hormone receptor signaling pathway, lipid metabolic process, and fatty acid oxidation are mainly metabolic disturbances of PCOS [44–46]. Identified PPDT-Module 2 had not only a good consistency function with PCOS but also good classification performance for classifying normal/PCOS samples and PCOS/after pioglitazone treatment samples before and after consistent check. These suggested that PPDT-Module 2 responded to drug treatment efficiently, may be used as an integral PCOS potential therapeutic target. 22 PCOS potential drug targets possessed central importance in PPDT-Module 2 as well as had good consistent function with PCOS. It was worth noting that 22 PCOS potential drug targets we identified were all interconnected in PPDT-Module 2, and had good classification performance, as same as one with PPDT-Module2, could represent the whole PPDT-Module 2 well.

Recent studies have demonstrated that the beneficial metabolic effects of treatment with pioglitazone induce adipogenesis and have insulin-sensitizing and antidiabetic properties in PCOS patients [52, 53]. It is known that different patients' response to the drug treatment are different, thus the efficacy of treatment is various. The consistent check could identify status-consistent samples to improve the identification accuracy of disease status and effectiveness evaluation accuracy of drug response. The classification performances of samples with consistent expressions were much better than that of all samples with the average expression values of PCOS potential drug targets, PPDT-Module 2, PCOS disease genes, T2D disease genes and known T2D drug targets as classification features to classify 3 statuses samples of the expression profile GSE8157, respectively (Figure 4). In the meantime, the classification performances of samples with consistent expressions were also much better than that of all samples of original expression profile with same classification features in another independent expression profile GSE6798 related to PCOS (Supplementary Figure S1). Interestingly, the classification performances of PCOS potential drug targets and PPDT-Module 2 as classification features were better than PCOS disease genes, T2D disease genes and known T2D drug targets in both expression profiles (Figure 5, Supplementary Table 5, Supplementary Table 6 and Supplementary Table 7).

The potential drug targets and disease genes were usually identified based on significantly differential expressed genes [54]. It was noted that between normal samples and PCOS samples no significantly differential expressed genes of expression profiles GSE8157 and GSE6798 could be identified, while between PCOS samples and after pioglitazone treatment samples of GSE8157 only few significantly differential expressed genes could be identified, including no known disease genes, known drug targets or our interested genes. So the potential drug targets and disease genes could not be identified from the point of differential expressed genes for these two expression profiles. Nevertheless, identified PPDT-Module 2 and 22 PCOS potential drug targets could distinguish the statuses between normal and disease, evaluate the effectiveness of drug treatment from the systems level and functional point. Just like our research on the PCOS from the biological background, previous researchers used the profile GSE8157 to identify differential pathways instead of differential expressed genes, for analyzing the pathogenesis of PCOS and the way of drug response [52]. It was shown that from the system level and biological background to study the disease statuses and drug response could obtain comprehensive and effective results, especially in the case of no significantly differential expressed genes.

In addition, 21 PCOS potential drug targets were verified by literatures to be associated with the pathogenesis and treatment of PCOS, which could reflect the status of disease and drug response. Drugs targeting these potential drug targets may be potential drugs for PCOS. And 42 drugs targeting to 13 PCOS potential drug targets were verified to be investigated experimentally or clinically for treating PCOS (Table 3). As a drug target, PPARG is widely studied and used for T2D, and is also researched to treat PCOS [55, 56]. There are two classes of drugs targeting to PPARG for T2D. One is insulin sensitization agent such as Pioglitazone, Rosiglitazone; the other is correcting the lipid metabolism disorders agent such as Repaglinide, Telmisartan. These drugs targeting to PPARG were highly possible to act as potential drugs for PCOS. Desogestrel, Ethinyl Estradiol, Clomifene and Tamoxifen are targeting to ESR1 (1st). Desogestrel as synthetic progestational hormone component and Ethinyl Estradiol as estrogenic component in oral contraceptives were used to improve clinical hyperandrogenism in PCOS in the first-line medicine [57, 58]. Clomifene is a triphenyl ethylene stilbene derivative as an estrogen agonist or antagonist, which is the most commonly used drug for first-line treatment of ovulation induction in women with PCOS [59–61].

**Table 3:** Drugs targeting PCOS potential drug targets

PCOS potential drug targets	Drugs
ESR1	Mestranol^b^, Fulvestrant^a^, Danazol^a^, Etonogestrel^b^, Raloxifene^b^, Diethylstilbestrol^b^, Medroxyprogesterone Acetate^b^, Genistein^a^, Desogestrel^b^, Norgestimate^b^, Naloxone^b^, Levonorgestrel^a^, Ethinyl Estradiol^b^, Melatonin^a^, Clomifene^b^, Progesterone^b^, Tamoxifen^b^, Estradiol^b^, Estrone^a^, Estriol^a^
RXRA	Alitretinoin^a^
NCOA1	Genistein^a^
ESR2	Raloxifene^b^, Diethylstilbestrol^b^, Genistein^a^, Tamoxifen^b^, Estradiol^b^
THRB	Levothyroxine^a^
RARA	Alitretinoin^a^, Isotretinoin^b^
PPARA	Gemfibrozil^a^, Bezafibrate^b^, Indomethacin^a^
PPARG	Repaglinide^a^, Glipizide^a^, Telmisartan^a^, Indomethacin^a^, Pioglitazone^b^, Rosiglitazone^b^, Bezafibrate^b^
PGR	Mifepristone^a^, Danazol^a^, Etonogestrel^a^, Megestrol acetate^b^, Medroxyprogesterone Acetate^b^, Desogestrel^b^, Norgestimate^b^, Levonorgestrel^a^, Drospirenone^b^, Spironolactone^b^, Norethindrone^b^, Progesterone^b^
ESRRG	Diethylstilbestrol^b^
RXRB	Alitretinoin^a^, Tretinoin^a^
RARG	Alitretinoin^a^, Tretinoin^a^
VDR	Alfacalcidol^a^, Calcidiol^a^, Ergocalciferol^a^, Cholecalciferol^a^, Calcitriol^a^

a denotes the drug is under experimental investigation;

b denotes the drug has been investigated to be used for treating to PCOS in clinical.

Some drugs are targeting several PCOS potential drug targets such as Alitretinoin targeting RXRA, RARA, RXRB and RARG; Tamoxifen targeting ESR1 and ESR2. Retinoid X receptors (RXRs) and retinoic acid receptors (RARs) are nuclear receptors that mediate the biological effects of retinoids by their involvement in retinoic acid-mediated gene activation. These receptors function as transcription factors by binding as homodimers or heterodimers to specific sequences in the promoters of target genes. The proteins encoded by these genes are members of the steroid and thyroid hormone receptor superfamily of transcriptional regulators [62]. Alitretinoin is also known as retinoic acid and derived from maternal vitamin A. Altered retinoic acid synthesis and action could influence the expression of these genes and androgen production in PCOS [63]. Tamoxifen, as one of the selective estrogen receptor modulators (SERM) with tissue-specific activities, is discovered to be a good alternative to clomiphene in women with PCOS and clomiphene-resistant case [64]. These durgs may play key roles in treating PCOS by multiple ways.

As shown in a series of recent publications [16, 65] in developing new findings and approaches, user-friendly and publicly accessible web-servers will significantly enhance their impacts [20], we shall make efforts in our future work to provide a web-server to displaying findings that can be manipulated by users according to their need.

Our approach was performed based on the existing known diseases genes and drug targets. As the disease genes and drug targets increased, our approach would be more precise. In the meantime, more and more gene expression datasets and novel drugs are available, which will make the result evaluation more objective and precise. The median of the Jaccard similarity indexes was selected as the threshold to identify the pathobiological similar modules in our study, which should be further optimized for the application of other diseases. In addition, the side-effect and toxicology of the drugs identified by identified PCOS potential drug targets were not considered adequately in our study, which will be further improved in our future work.

We proposed a new computational approach to identify the PPDT-Module and PCOS potential drug targets from the systems level and biological background based on the PPIN and pathobiological similarity. 21 genes of 22 identified PCOS potential drug targets were verified to be associated with the pathogenesis of PCOS, and 42 drugs targeting 13 PCOS potential drug targets were investigated experimentally or clinically for PCOS. These PCOS potential drug targets and PPDT-Module could reveal the drug response, distinguish the statuses between normal and disease, and act as PCOS biomarkers. Moreover, further research are expected for the ways of drug response. Our study would shed light on the treatment of PCOS, and provide new insights to research on the pahogenesis and drug response of other diseases that possess high pathobiological similarity.

## MATERIALS AND METHODS

### Data source

The Protein-Protein Interaction Network was obtained from the Human Protein Reference Database (http://www.hprd.org/) [66], which contained 37041 protein-protein interaction pairs between 9518 proteins. Our research was based on two gene expression profiles from the Gene Expression Omnibus (GEO) database (http://www.ncbi.nlm.nih.gov/geo/) [67] : GSE8157 including 33 samples (13 control samples and 10 PCOS samples before and after pioglitazone treatment, respectively) and GSE6798 including 29 samples (13 control samples and 16 PCOS samples) in GPL570. 35 PCOS disease genes and 83 T2D disease genes were extracted from GAD (http://geneticassociationdb.nih.gov/) [68], DO (http://www.disease-ontology.org/) [69] and OMIM (http://www.ncbi.nlm.nih.gov/omin) [70]; 62 T2D drug targets were extracted from Drug Bank (http://www.drugbank.ca/) [70].

### Identification of PPDT-Modules

We put forward a new approach to identify PPDT-modules in the PPIN based on the pathobiological similarity of PCOS and T2D. (i) PCOS disease genes and T2D disease genes were set as seeds to filter the no weight protein-protein interaction network and obtained the disease genes related no weight 3-step-neighbor sub-network, respectively. T2D and PCOS related modules (T-Modules and P-Modules) were mined using the Markov Cluster Algorithm (MCL Algorithm) in the 3-step-neighbor sub-network (no requirement of minimum number of seeds each cluster needs to contain), respectively. The MCL algorithm is a fast and scalable unsupervised cluster algorithm for networks, resulting in a collection of densely connected groups of genes [71]. The Markov clustering parameter was set to the default value 1.8. (ii) The pathobiological similar modules were defined as the intersection of P-Module and T-Module pairs, if their Jaccard similarity indexes were larger than their median, and their intersection contained the PCOS disease genes and T2D disease genes simultaneously. The Jaccard similarity index J(P,T) reflected the consistency of each two modules P-Module and T-Module:

$$\text{J}\left(\text{P},\text{T}\right)=\frac{\left|P\cap T\right|}{\left|P\cup T\right|}$$ (1)

where P is a P-Module and T is a T-Module. The numerator is the number of common genes in P and T. The denominator is the number of all genes in P and T. (iii) Candidate PPDT-Modules were the pathobiological similar modules that included T2D drug targets, and PPDT-Modules were the candidate modules which possessed consistent function with PCOS.

### Analysis of the central importance of genes in PPDT-Modules

The genes possessing more neighbors, more paths through them and so on, could play the central role in PPDT-Modules. Hence, the G-rank, G_gi_, was used to evaluate the central importance of gene g_i_ in these modules, which integrated 4 topological features, degree, betweenness, closeness and page rank, utilizing the geometric mean rank:

$${\text{G}}_{\text{gi}}={\text{Rank}}_{\text{ascending}}{\left(\prod_{j=1}^{4}{\text{R}}_{\text{gij}}\right)}^{\frac{1}{4}}$$ (2)

where Rg_ij_ is the rank of g_i_ for the feature j(degree, betweenness, closeness and page rank when j = 1, 2, 3, 4, respectively).

The degree of g_i_ is the number of edges linked to g_i_.

The closeness of g_i,_ C_gi_, is defined as the inverse of the average geodesic distance (or length of the shortest path) to all other genes:

$${\text{C}}_{\text{gi}}={\left(\frac{1}{\left(n-1\right)}\sum_{g_{p}}\neq {\;}_{\text{gi}}d_{g_{p}\text{ gi}}\right)}^{-1}$$ (3)

where $d_{g_{p}g_{i}}$ denotes the length of shortest path between g_i_ and g_p_, n denotes the number of all genes in the module.

The betweenness of gi, B_gi_, indicates how many of the shortest paths between any two genes in the network include g_i_:

$${\text{B}}_{\text{gi}}=\frac{1}{\left(n-1)(n-2\right)}\sum_{g_{p}\neq g_{q}\neq g_{i}}\frac{\sigma_{g_{p}g_{q}g_{i}}}{\sigma_{g_{p}g_{q}}}$$ (4)

where $\sigma_{g_{p}g_{q}g_{i}}$ denotes the number of the shortest path between g_p_ and g_q_ through g_i_, and $\sigma_{g_{p}g_{q}}$ denotes the number of the shortest paths between g_p_ and g_q_.

The page rank of g_i,_ PR_gi_, should be highly ranked if other highly ranked genes contained edges to it:

$$PR_{\text{gi}}=\text{d}\sum_{g_{p}\neq g_{i}}\frac{L\left(g_{p}\right)}{PR\left(g_{p}\right)}+\frac{1-d}{n}$$ (5)

where PR(g_p_) denotes the rank of g_p_, L(g_p_) denotes the number of edges of g_p_, d ∈ (0, 1) is a fixed parameter.

### Identification and analysis of PCOS potential drug targets

The PCOS potential drug targets were defined as the genes with high G-ranks (upper quartile) in PPDT-Modules, as well as correspond to the function of PCOS disease genes (FDR *p* < 0.05). The functional annotation and literature verification were conducted to investigate the correlation between PCOS potential drug targets and PCOS. The functional annotation analysis was performed with all genes in PPDT-Modules and PCOS disease genes using the hyper-geometric test:

$$P_{\left(X=k\right)})=\frac{C_{M}^{k}C_{N-M}^{n-k}}{C_{N}^{n}}$$ (6)

where N denotes the number of all human genes, n denotes the number of genes of PPDT-Modules and PCOS disease genes, M denotes the number of genes in function i, k denotes the number of genes of PPDT-Modules and PCOS disease genes in function i. The False Discovery Rate (FDR) method was used to correct *p*-value, and *p*-value < 0.05 was set as the criterion for this analysis [72–74].

### Efficiency analysis of PCOS potential drug targets

To assess the efficiency of PCOS potential drug targets, the average expression values of them were used as input classification feature values for an independent expression profile of drug treatment (GSE8157, PCOS samples before and after pioglitazone treatment and normal samples) using Support Vector Machine (SVM). In machine learning, SVM is a supervised learning model with associated learning algorithms that analyze data and recognize patterns, which is used for classification and regression analyses [75]. The jackknife test has been increasingly used by investigators to test the power of various predictors, however, to reduce the computational time, we adopted the five-fold cross-validation as done by many investigators using SVM as the prediction engine [16, 65]. A receiver operating characteristic (ROC) curve was plotted and the area under the curve (AUC), the sensitivity, specificity, accuracy and MCC score were calculated. The five-fold cross-validation was applied 1000 times, and the 1000 ROC curves and AUC scores were obtained to evaluate the robustness of the PCOS potential drug targets. The higher the AUC score was, the better the classification performance was.

## SUPPLEMENTARY MATERIALS FIGURE AND TABLES


