# Supplementary material for: Identification of polycystic ovary syndrome potential drug targets based on pathobiological similarity in the protein-protein interaction network
Source: Oncotarget. 2016 May 13;7(25):37906–19. doi: 10.18632/oncotarget.9353 (PMC5122359; doi:10.18632/oncotarget.9353)
Supplement: Supplementary file 1 [file oncotarget-07-37906-s001.pdf]

Identification of polycystic ovary syndrome potential drug targets based on pathobiological similarity in the protein-protein interaction network

Supplementary Materials

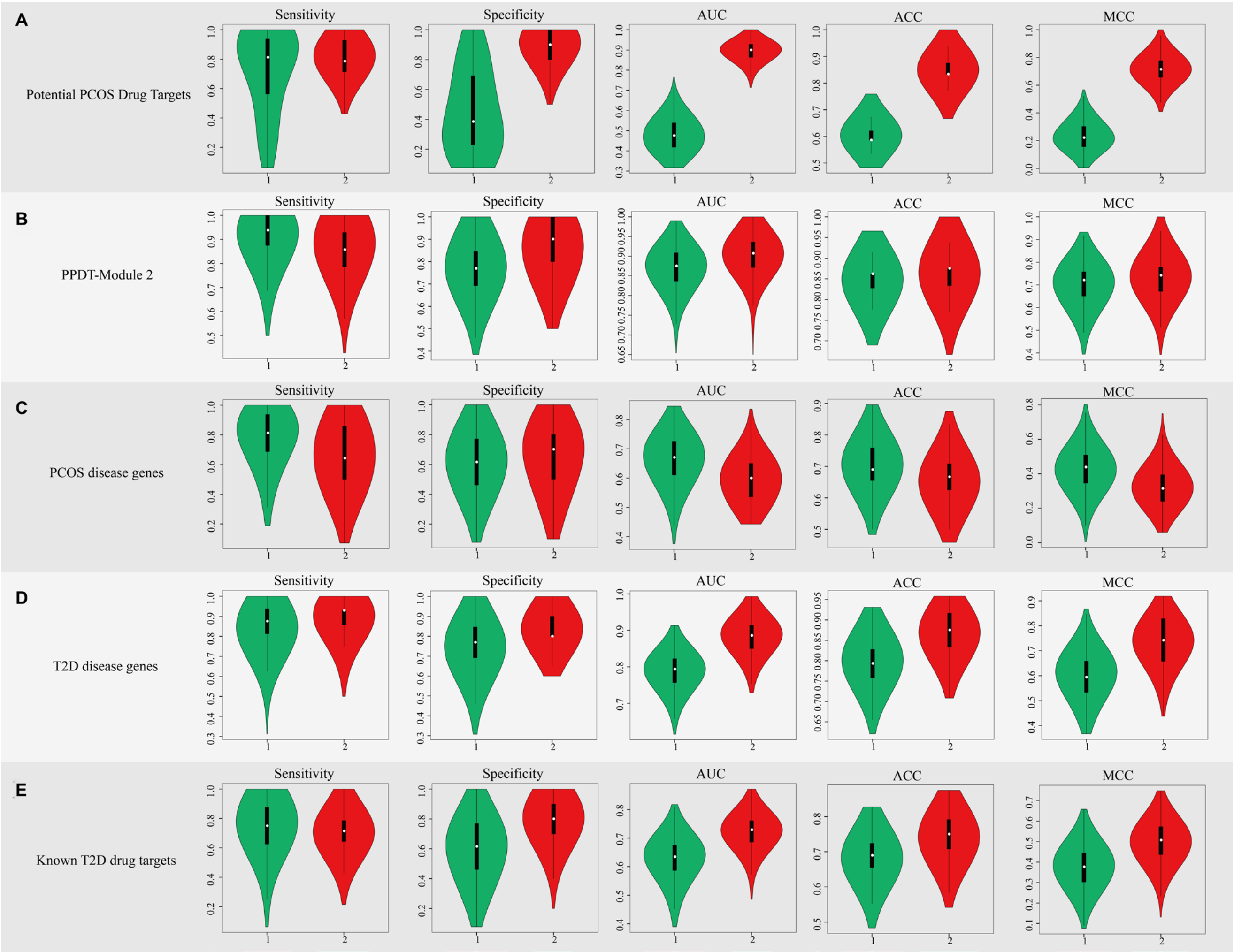

Supplementary Figure S1: The sensitivity, specificity, AUC, ACC and MCC score distribution of different classification features with 1000 times five-fold cross-validation for GSE6798. The green represent the classification of the samples before consistency check, the red represents the classification of the samples after consistency check.

Supplementary Table S1: Genes of candidate PPDT-Modules

Candidate PPDT-Module	Genes
PPDT-Module 1	DCAF7, SRRM1, DENND4A, KIF1C, SRSF10, PAPOLA, YWHAQ, CDC42EP1, RASSF8, PARD3B, CLK1, CLK2, CLK3, FRMD6, HSPB6, PDCL2, KLC3, SIK1, CTPS1, ADRA2B, ADRA2C, JAKMIP1, DHX15, DYNC1H1, DYRK1A, PGAM5, EIF2B1, CRTC2, MARK2, CCNY, LPHN1, MAPRE1, MAPRE3, STK38L, SAMD4A, FRYL, MYCBP2, N4BP3, MPRIP, TBC1D1, SIK2, ANKS1A, KIAA0930, FMR1, CLASP1, LARP1, CRTC1, SRGAP2, SIK3, TDRD7, SRRM2, GTPBP4, SH3BP4, FUS, EML3, HECTD1, OSBPL3, RAI14, GAPVD1, CKAP2, TMEM102, CFAP20, TRA2A, PIK3R4, TLX2, IGSF1, SHROOM2, KCNK3, KIF5A, KIF5B, KIF5C, INSC, MFAP1, PPP1R12A, CDK17, KCNK9, LIMA1, LSR, LUC7L3, CDK14, SLC25A3, PIK3C3, PI4KB, MIEF1, PLEKHA5, SAMD4B, RALGPS2, PRPF38B, RMDN3, PPP6R3, PPP3CB, FGD6, BAIAP2L1, MAP2K5, INPP5E, CGN, NUFIP2, ALS2, USP37, PTPN1, KCNK15, CRTC3, WNK1, SLC8A2, SLC8A1, SLC8A3, SOD1, SON, TH, UCP2, UCP3, YWHAB, YWHAE, YWHAG, YWHAH, YWHAZ, SF1, ZFP36, MAPKAP1, EDC3, SH3BP5L, NDEL1, CCNL2, RIOK1, STK24, LRCH3, MICALL1, DOCK7, GBF1, PRPF4B, PHLDB2, DYRK1B, ARHGEF2, SHKBP1, PPIG, RASAL2, KIF23, FARP2, CEP170, THRAP3, CCS
PPDT-Module 2	BCAS2, SLC30A9, DDX17, PPARGC1A, PNRC1, PPARGC1B, NCOA7, DDX5, DUT, NR0B1, AHR, ESR1, ESR2, ESRRA, ESRRG, FABP1, ARHGEF15, MTCH2, STAC3, NR5A2, TRIM59, MKNK2, ATAD2, PSMC3IP, LINC00312, NRBF2, HNF4A, HNF4G, PRMT2, MAP6, NR3C2, MPG, IRX4, MECR, PGR, BCL11A, POU4F2, PPA1, PPARA, PPARD, PPARG, MED1, KIF1A, ZCCHC10, WIPI1, PNRC2, PROX1, PAK6, REXO4, ZNF398, RARA, RARB, RARG, KDM5A, RORA, RORB, RXRA, RXRB, RXRG, MMS19, SMARCD3, KLF9, TCF20, NR2F1, NR2F2, THRB, NR2C1, VDR, XBP1, CUEDC2, DDX54, CHD9, NCOA3, NRIP1, NRIP2, NR0B2, HELZ2, ACTL6A, NCOA1, EDF1, HMGN3, ZNHIT3, RBM39, MED24, NR1I3, NR1H4
PPDT-Module 3	KRT27, IGF1R, INSR, INSRR, ENPP1, KL

Supplementary Table S2: G-rank of genes of PPDT-Module 2

Gene of PPDT-Module2	G-rank	Ranks of Degree	Ranks of Betweenness	Ranks of Closeness	Ranks of Page Rank
ESR1	1	1	1	1	1
RXRA	2	2	2	2	2
NCOA1	3	3	3	3	3
NRIP1	4	5	8	4	6
ESR2	5	4	7	12	4
THRB	6	6	10	8	5
RARA	7	8	5	9	7
NR0B2	8	9	12	5	11
NCOA3	9	10	13	6	9
HNF4A	10	11	9	11	12
PPARA	11	12	4	30	10
PPARG	12	7	11	28	8
PPARGC1A	13	13	22	7	13
MED1	14	14	20	10	14
NR2F1	15	28	6	13	24
PNRC2	16	15	19	16	17
PGR	17	19	14	22	15
ESRRA	18	18	21	14	19
ESRRG	19	16	23	39	16
RXRB	20	17	35	27	18
RARG	21	20	26	33	23
VDR	22	21	25	36	21
SMARCD3	23	29	28	15	33

PNRC1	24	23	24	54	20
PROX1	25	24	15	70	25
NR0B1	26	32	34	19	32
RXRG	27	22	30	50	22
NR1H4	28	31	39	18	34
PRMT2	29	33	44	17	30
PPARD	30	26	27	43	27
RARB	31	27	32	38	28
DUT	32	37	31	23	36
MED24	33	41	37	20	45
CHD9	34	42	36	21	44
NR5A2	35	25	33	68	26
RORB	36	30	29	65	29
WIPI1	37	56	18	44	39
DDX5	38	39	40	29	40
AHR	39	40	51	24	43
NRBF2	40	36	42	46	37
RORA	41	35	38	69	31
MECR	42	38	43	49	38
BCL11A	43	61	16	77	41
NR1I3	44	34	47	56	35
KIF1A	45	57	45	25	54
STAC3	46	62	17	81	46
KDM5A	47	53	46	34	51
PPARGC1B	48	51	49	35	52
MMS19	49	45	64	32	50
RBM39	50	47	56	40	47
MPG	51	52	71	26	55
BCAS2	52	48	57	41	48
MAP6	53	43	41	73	42
DDX54	54	50	58	42	49
PSMC3IP	55	46	55	55	56
EDF1	56	49	48	66	57
ATAD2	57	58	63	37	68
ZNHIT3	58	44	54	67	59
ARHGEF15	59	68	50	45	67
NR2C1	60	67	77	31	69
NCOA7	61	60	66	47	63
HNF4G	62	55	53	72	58
DDX17	63	54	62	71	53
MKNK2	64	63	70	48	64
CUEDC2	65	65	72	53	60
REXO4	66	66	73	51	65
NRIP2	67	64	52	74	70
HELZ2	68	59	59	78	71
ZNF398	69	72	65	57	76
TRIM59	70	71	85	52	66

TCF20	71	73	67	58	77
PAK6	72	74	68	59	78
HMGN3	73	70	61	75	73
FABP1	74	69	60	79	72
XBP1	75	75	69	60	79
LINC00312	76	76	74	61	80
NR2F2	77	81	80	84	61
SLC30A9	78	83	82	62	81
ZCCHC10	79	82	81	85	62
MTCH2	80	84	83	63	82
PPA1	81	79	78	82	74
KLF9	82	80	79	80	75
POU4F2	83	85	84	64	83
IRX4	84	78	76	76	85
NR3C2	85	77	75	83	84

Supplementary Table S3: Part of functional annotation results of PPDT-Module 2 and PCOS disease genes

Category	Function Term	Number of Genes	FDR *P* value
BP	GO:0045449~regulation of transcription	70	7.92E–24
BP	GO:0006350~transcription	60	7.36E–20
BP	GO:0030518~steroid hormone receptor signaling pathway	14	3.55E–13
BP	GO:0030521~androgen receptor signaling pathway	7	3.91E–04
BP	GO:0030520~estrogen receptor signaling pathway	5	0.001497484
BP	GO:0019216~regulation of lipid metabolic process	9	0.002953386
BP	GO:0046321~positive regulation of fatty acid oxidation	4	0.035525241
MF	GO:0003707~steroid hormone receptor activity	30	6.77E–48
MF	GO:0004879~ligand-dependent nuclear receptor activity	31	3.51E–47
MF	GO:0005496~steroid binding	16	6.13E–16
MF	GO:0008289~lipid binding	23	1.82E–09
MF	GO:0035258~steroid hormone receptor binding	10	8.74E–09
MF	GO:0030331~estrogen receptor binding	7	3.16E–07
MF	GO:0050681~androgen receptor binding	6	0.001572181
KEGG	hsa03320:PPAR signaling pathway	7	0.014854388
KEGG	hsa00140:Steroid hormone biosynthesis	6	0.027319408

Supplementary Table S4: Literature verification of PCOS potential drug targets associated with pathogenesis of PCOS

PCOS potential drug targets	Pubmed ID
ESR1	PMID: 25617525
RXRA	PMID: 15914525
NCOA1	PMID: 12050280
NRIP1	None
ESR2	PMID: 21824047
THRB	PMID: 26339875
RARA	PMID: 15914525
NR0B2	PMID: 24517280
NCOA3	PMID: 16677694

HNF4A	PMID: 22904171
PPARA	PMID: 16192401
PPARG	PMID: 24649096
PPARGC1A	PMID: 20130411
MED1	PMID: 26339875
NR2F1	PMID: 25617525
PNRC2	PMID: 15970482
PGR	PMID: 25750105
ESRRA	PMID: 26649621
ESRRG	PMID: 26649621
RXRB	PMID: 15914525
RARG	PMID: 15914525
VDR	PMID: 26458343

Supplementary Table S5: Average classification performance with different features for normal/PCOS samples of GSE8157

**Samples**	**Normal/PCOS**									
**Consistency check**	**Before consistency check**					**After consistency check**				
Performance	Sn	Sp	AUC	ACC	MCC	Sn	Sp	AUC	ACC	MCC
PCOS potential drug targets	0.68	0.79	0.72	0.73	0.49	0.88	0.96	0.91	0.92	0.84
PPDT-Module 2	0.83	0.93	0.92	0.87	0.77	0.99	0.97	0.98	0.98	0.96
PCOS disease genes	0.70	0.84	0.76	0.76	0.55	0.72	0.96	0.79	0.84	0.71
T2D disease genes	0.65	0.88	0.73	0.75	0.54	0.93	0.95	0.95	0.94	0.89
Known T2D drug targets	0.702	0.95	0.79	0.81	0.66	0.86	0.95	0.88	0.91	0.82

Supplementary Table S6: Average classification performance with different features for PCOS/after pioglitazone treatment samples of GSE8157

**Samples**	**PCOS/after pioglitazone treatment**									
**Consistency check**	**Before consistency check**					**After consistency check**				
Performance	Sn	Sp	AUC	ACC	MCC	Sn	Sp	AUC	ACC	MCC
PCOS potential drug targets	1	1	1	1	1	1	1	1	1	0.85
PPDT-Module 2	0.98	0.99	0.99	0.99	0.98	0.99	1	0.99	0.99	0.81
PCOS disease genes	0.99	0.99	0.99	0.99	0.99	0.99	1	0.99	0.99	0.82
T2D disease genes	0.98	0.99	0.99	0.99	0.97	0.99	1	0.99	0.99	0.84
Known T2D drug targets	0.99	0.99	0.99	0.99	0.99	0.99	1	0.99	0.99	0.81

Supplementary Table S7: Average classification performance with different classification features of GSE6798

**Samples**	**Normal/PCOS**									
**Consistency check**	**Before consistency check**					**After consistency check**				
Performance	Sn	Sp	AUC	ACC	MCC	Sn	Sp	AUC	ACC	MCC
PCOS potential drug targets	0.73	0.45	0.48	0.60	0.23	0.81	0.90	0.89	0.85	0.72
PPDT-Module 2	0.90	0.78	0.87	0.85	070	0.87	0.85	0.90	0.86	0.74
PCOS disease genes	0.78	0.61	0.66	0.70	0.42	0.65	0.65	0.60	0.65	0.33
T2D disease genes	0.84	0.74	0.79	0.80	0.60	0.89	0.83	0.88	0.87	0.73
Known T2D drug targets	0.75	0.59	0.63	0.68	0.38	0.70	0.79	0.72	0.73	0.50
